# Characterization and whole genome sequencing of a novel strain of *Bergeyella cardium* related to infective endocarditis

**DOI:** 10.1186/s12866-020-1715-0

**Published:** 2020-02-12

**Authors:** Hongwei Pan, Wei Li, Enhua Sun, Yi Zhang

**Affiliations:** Department of Clinical Laboratory, Qilu Hospital of Shandong University, Jinan, 250012 Shandong Province China

**Keywords:** *Bergeyella cardium*, Infective endocarditis, Genome sequencing

## Abstract

**Background:**

*Bergeyella cardium* infection is becoming increasingly prevalent in patients with infective endocarditis, suggesting its significance in disease pathogenesis. However, few studies have fully characterized this species.

**Results:**

Herein, we report the morphological and physiological characteristics, as well as whole genome sequencing of a newly identified *B. cardium* HPQL strain isolated from a patient with infective endocarditis. Results from the cellular morphology and biochemical analysis provide basic knowledge on the new pathogen. The whole genome sequencing of *B. cardium* HPQL consists of a circular chromosome with a total length of 2,036,890 bp. No plasmid was detected. Comparative genomics were carried out then. Antibiotics resistance related genes, pathogenesis related genes, predicted insertion sequences, genome islands and predicted CRISPRs sequences were demonstrated. To our knowledge, this is the first study to provide a complete genome sequence for *Bergeyella* spp.

**Conclusions:**

This study provides fundamental phenotypic and genomic information for the newly identified fastidious infective endocarditis causative bacteria, *B. cardium*. Our results provide insights into effective clinical diagnosis and treatment of this pathogen.

## Background

Infective endocarditis is a serious infectious disease with high associated morbidity and mortality. Identification of the causative agents is, therefore, crucial for improving the clinical outcome [1]. Clinically, infective endocarditis is generally diagnosed based on positive blood cultures, removed leads, and/or infected pocket material [1]. Species belonging to the genera *Streptococcus*, *Staphylococcus*, and *Enterococcus* are the primary causative organisms of infective endocarditis [2]. However, recently new pathogens are emerging as additional etiological agents, such as *Bergeyella* spp. [3–5].

*Bergeyella* spp. are non-fermenting gram-negative bacilli, belonging to the family *Flavobacteriaceae* [3]. *B. zoohelcum*, known to cause cellulitis, leg abscess, tenosynovitis, septicemia, pneumonia, and meningitis, is one of the best described zoonotic pathogens afflicting humans [6, 7]. *B. zoohelcum* is usually isolated from the normal oral microflora of animals such as cats and dogs [8]. Hence, animal bites and prolonged exposure to pets are the primary causes of human infection with *B. zoohelcum* [4]. In addition, a patient suffered *B. zoohelcum* bacteremia after eating food prepared with coagulated goat blood [9, 10]. A case of cellulitis due to *B. zoohelcum* infection was also reported in a tsunami victim [9]. Alternatively, infections caused by other *Bergeyella* spp. are rarely reported. A previously uncultivated *Bergeyella* sp. (clone AF14) with strong homology to a previously reported uncultivated oral *Bergeyella* strain was suspected to be an opportunistic pathogen during preterm birth [11]. Further, the isolation of two *Bergeyella* strains was reported from patients with infective endocarditis. Both strains shared 94.9% homology with *B. zoohelcum*, suggesting that they are a new species belonging to of the genus Bergeyella. The two strains were designated as *Bergeyella cardium*13-07^T^ and *Bergeyella cardium*13–16 [3]. Meanwhile, another case study reported the isolation of a *Bergeyella* strain from an infective endocarditis patient that had 98.2% shared identity with *B. zoohelcum*, which was slightly lower than the ≥99.0% homology required for two organisms to be considered the same species [4]. Recently, a novel *Bergeyella* sp. was isolated from a patient with infective endocarditis. The organism was determined to be genetically most closely related to *B. cardium* [12]. Moreover, the first case of *B. cardium* prosthetic valve endocarditis was also reported quite recently [5].

The increasing number of cases of *B. cardium* infection in patients with infective endocarditis suggests its importance in disease pathogenesis. However, studies examining the microbial characteristics and genetic features of this species are very rare. In this study, we therefore, sought to describe the isolation, identification and characterization of a new *B. cardium* sp. from blood cultures of a patient with infective endocarditis. We also performed whole genome sequencing and, through phylogenetic analysis, we were able to predict the possible origin of this newly identified species.

## Results

### Phylogenetic analysis identified the isolate as a novel species of *B. cardium*

**Fig. 1:**
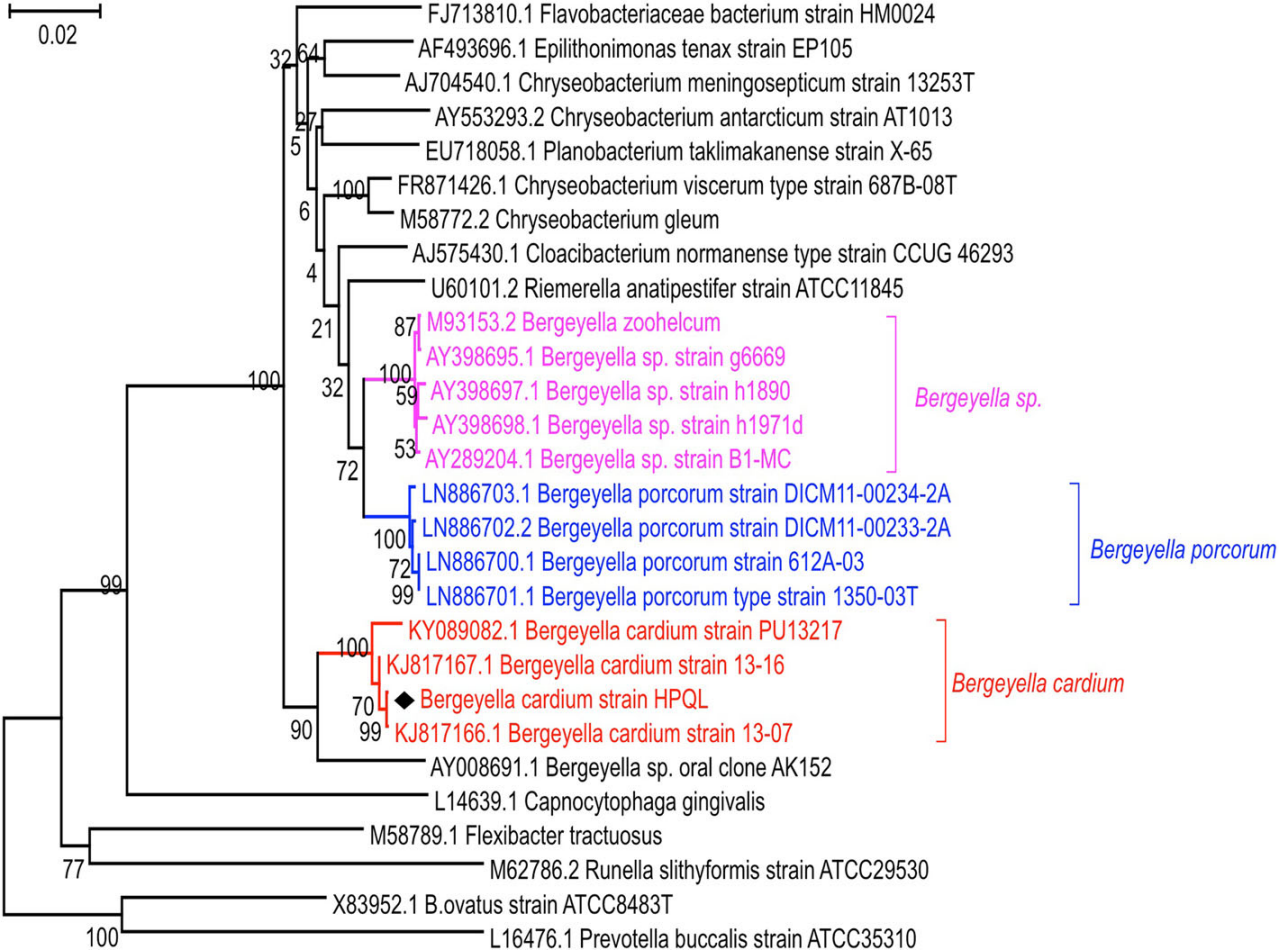
Phylogenetic tree based on 16S rRNA gene sequences. Phylogenetic analysis was performed on *B. cardium* HPQL and closely related species based on 16S rRNA gene sequences. The HPQL strain was determined to cluster with *B. cardium* spp. The phylogenetic tree was created using the Neighbor-Joining algorithm. The branches are scaled in terms of the expected number of substitutions per site

Four days after the initial blood culture was obtained from a 63-year-old man with infective endocarditis, the growth of microorganisms was reported through an automated blood culture system. Gram staining revealed the presence of gram-negative bacilli. However, no reliable identification was made by matrix-assisted laser desorption/ionization-time of flight mass spectrometry (MALDI-TOF MS). Thus, to accurately identify the pathogenic species, a 1425 bp sequence from the 16S rRNA gene of the isolated strain was amplified and sequenced via polymerase chain reaction (PCR). The sequence was then submitted to NCBI BLASTN to identify matched bacterial sequences. Results revealed that the sequence with the highest homology (approximately 99%) was isolated from *B. cardium*. To further verify the nucleotide BLAST results, a detailed phylogenetic tree was constructed as shown in Fig. 1. Results showed the isolated strain clustering with two *B. cardium* strains that had been isolated from two infective endocarditis patients in Korea [3]. Hence, the phylogenetic analysis supported the finding that the isolated strain was a novel strain of *B. cardium*. We, therefore designated the isolated strain in our study as *B. cardium* HPQL (identified by Hongwei Pan from QiLu hospital).

### General microbial characteristics of *B. cardium* HPQL

**Fig. 2:**
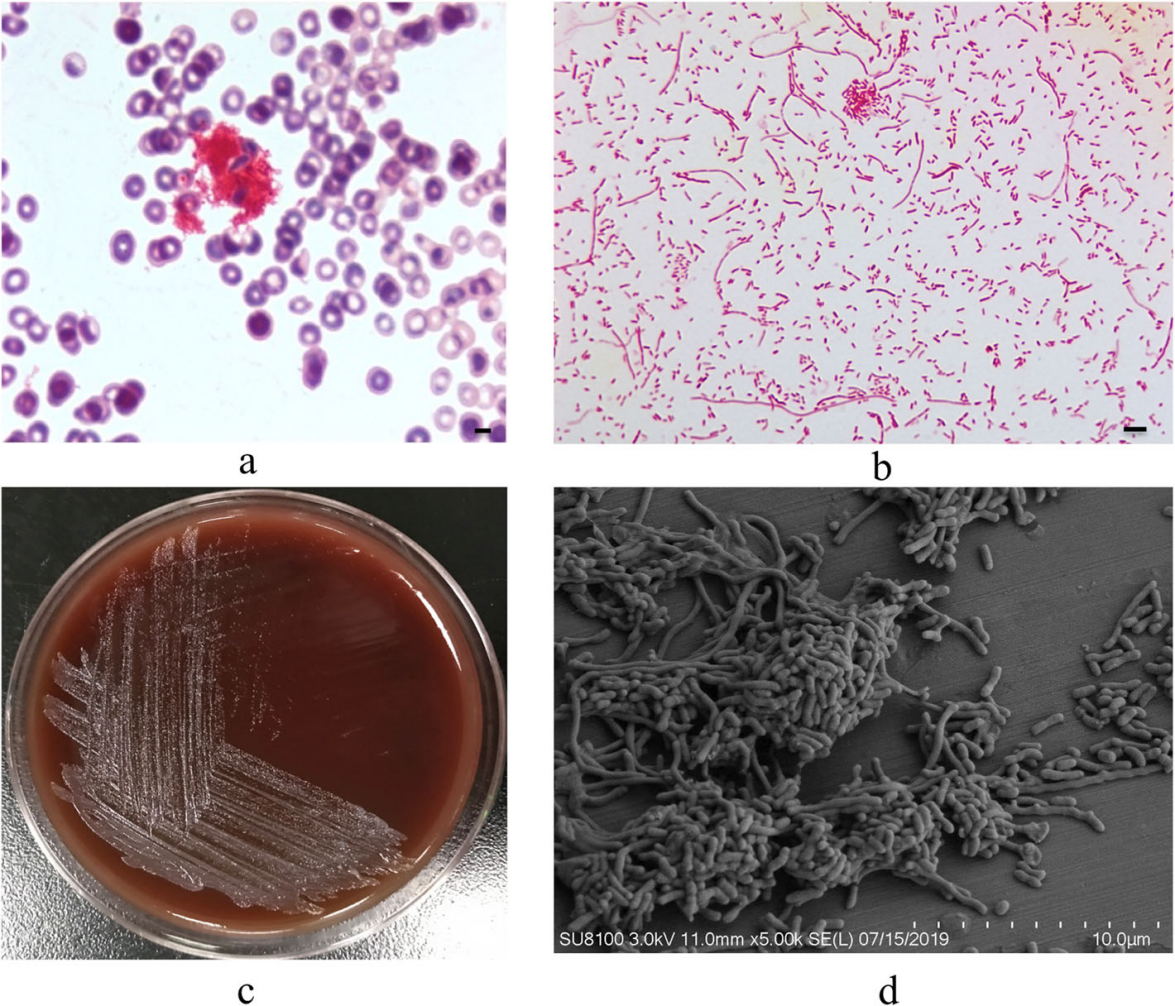
Morphological characterization of *B. cardium* HPQL. **a** Bacterial cells cultured from positive blood cultures. The bar is equal to 5 μm. **b** Gram staining properties of the HPQL strain. The bar is equal to 20 μm. **c** Bacterial colonies after culturing for 48 h on Columbia blood agar. **d** Scanning electron microscope observation of the bacterial cells

Morphological, physiological and biochemical characterization of the newly isolated strain was performed. We observed that the bacterial cells aggregate together in blood cultures (Fig. 2a). Moreover, small colonies of *B. cardium* HPQL were observed on blood agar after 48 h of incubation at 35 °C (Fig. 2c). However, the strain did not grow on MacConkey agar or Mueller-Hinton agar plates, indicating that this organism exhibits fastidious growth patterns. Further, individual colonies grown on blood agar appeared as non-pigmented, circular, shiny, and smooth with entire edges (Fig. 2c). Microscopic and scanning electron microscope observation revealed irregularly rod-shaped bacterial cells (Fig. 2b and d).

Biochemical characteristic of the strain were further analyzed. According to the results procured from API NH, this bacterial strain did not produce penicillinase, omithine decarboxylase, urease, β-galactosidase, proline arylamidase or gamma glutamyl transferase. However, it was positive for lipase, alkaline phosphatase and oxidase activity, and negative for catalase activity and indole production. Additionally, acid was found to be produced from D-glucose, D-fructose, D-maltose, D-sucrose.

### Antimicrobial susceptibility analysis

The E-test method was ultimately selected for AST analysis. MICs were determined following 4 days of growth on Columbia blood agar plates. The MICs for many of the selected antibiotics were quite low, with the exception of fluoroquinolones, chloramphenicol, azithromycin and gentamycin (Table 1).

**Table 1:** Minimum inhibitory concentration of antimicrobial agents of *B. cardium* HPQL

Antimicrobial agent (μ g/mL)	MICs after 96 h incubation
Penicillin	0.032
Ceftriaxone	0.048
Cefepime	0.016
Cefotaxime	0.024
Meropenem	0.012
Imipenem	0.032
Tigecycline	0.032
Amoxicillin/Clavulafiate	0.016
Sulfamethoxazole	0.094
Levofloxacin	0.5
Ciprofloxacin	0.5
Chloramphenicol	3
Azithromycin	8
Gentamycin	64

### Genomic features of the *B. cardium* HPQL strain

**Fig. 3:**
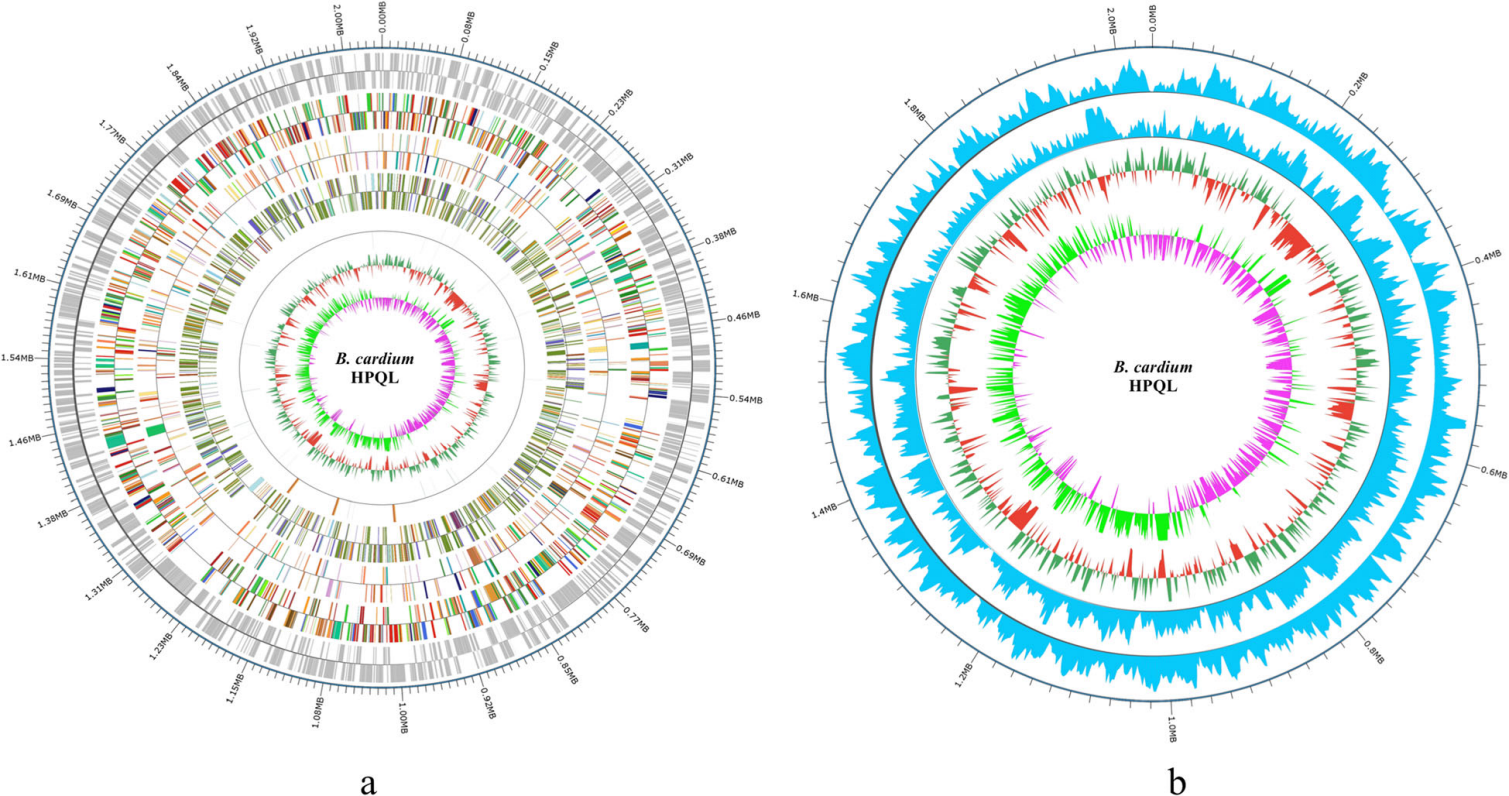
Chematic map of the *B. cardium* HPQL genome and distribution map of epigenetic modification. **a** Chematic map of the *B. cardium* HPQL genome. From outside, Circle 1: genome positions in kb; Circle 2: predicted protein coding sequences (CDSs) on the forward (outer wheel) and the reverse (inner wheel) strands; Circle 3, Circle 4 and Circle 5: gene annotation results, colored according to COG, KEGG, GO classification, respectively; Circle 6: ncRNA; Circle 7: GC content and GC content deviations from the average. **b** Distribution map of epigenetic modification. From outside, Circle 1: genome positions in kb; Circle 2: modification in sense strand; Circle 3: modification in antisense strand; Circle 4: GC content and GC deviations from the average

The whole genome of the newly isolated *B. cardium* HPQL strain was sequenced due to its potential clinical importance in patients with infective endocarditis. The genomic details are provided in Table 2 and Fig. 3a. Briefly, *B. cardium* HPQL contained a circular chromosome with a total length of 2,036,890 bp. The GC-content of the chromosome was determined to be 39.63%. The whole genome sequence contained 1896 predicted coding sequences (CDS), including 9 rRNAs, 42 tRNAs and 1 sRNA. A total of 1,813,065 predicted coding sequences were identified, which occupied 89.01% of the whole genome sequence. Additionally, 70.25% (1332/1896) of the protein-coding genes were assigned putative functions in the COG database, while the remaining genes were annotated as encoding hypothetical proteins. The distribution of genes in COGs functional categories are presented in Table 3. In addition, the methylation data of the whole genome are listed in Additional file 3 (Sheet S1), Fig. 3b and deposited to REBASE database.

**Table 2:** Statistics of *B. cardium* HPQL genome

Attribute	Value	% of total
Genome size (bp)	2,036,890	100%
%GC content of genome	39.63%	
Gene number	1896	100%
Gene length	1,813,065	89.01%
Gene Average Length	956	
Genes with function prediction	1332	70.25%
Genomic Island Numbers	3	0.16%
Genomic Island total Length	64,729	3.18%
No. of tRNA genes	42	2.22%
No. of rRNA operons	9	0.47%
No. of sRNA molecules	1	0.05%

**Table 3:** The genes of *B. cardium* HPQL genome in COG functional categories

Functional_class	Class_description	Gene_number
C	Energy production and conversion	87
D	Cell cycle control, cell division, chromosome partitioning	25
E	Amino acid transport and metabolism	89
F	Nucleotide transport and metabolism	52
G	Carbohydrate transport and metabolism	46
H	Coenzyme transport and metabolism	87
I	Lipid transport and metabolism	56
J	Translation, ribosomal structure and biogenesis	166
K	Transcription	51
L	Replication, recombination and repair	77
M	Cell wall/membrane/envelope biogenesis	148
N	Cell motility	12
O	Posttranslational modification, protein turnover, chaperones	82
P	Inorganic ion transport and metabolism	63
Q	Secondary metabolites biosynthesis, transport and catabolism	19
R	General function prediction only	98
S	Function unknown	62
T	Signal transduction mechanisms	37
U	Intracellular trafficking, secretion, and vesicular transport	23
V	Defense mechanisms	44
X	Mobilome: prophages, transposons	8

### Pathogenic analysis of *B. cardium* HPQL

A whole genome BLAST search was performed against the CARD, VFDB, and PHI databases to identify genes related to antibiotic resistance and virulence factors in the genome of *B. cardium* HPQL. Twelve genes were identified homology to well-known antimicrobial resistance genes (Additional file 4 Sheet S2). Moreover, a total of 70 genes related to putative virulence factors were identified in the genome of *B. cardium* HPQL (Additional file 5 Sheet S3), while 92 genes were described that may participate in bacteria-host interactions (Additional file 6 Sheet S4).

### Comparative genomic analysis of *B. cardium*

**Fig. 4:**
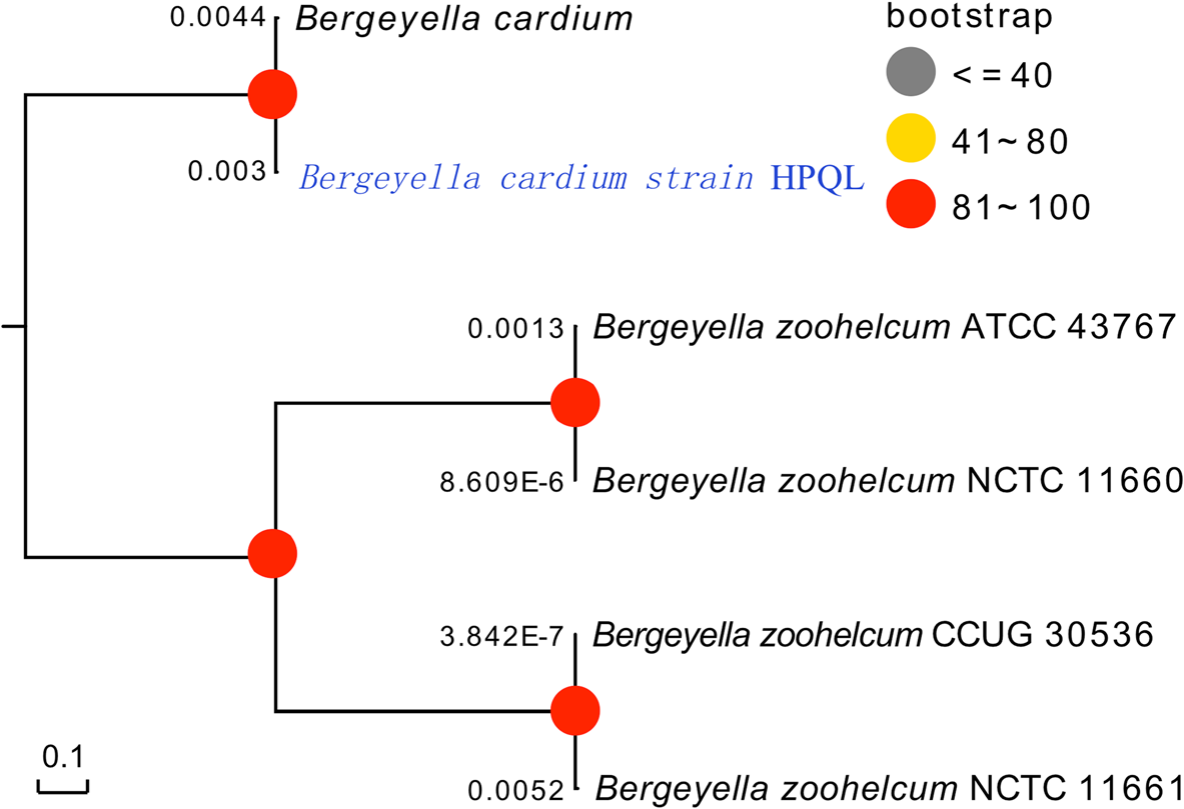
Comparative genomic analysis. Comparative genomic analysis between the *B. cardium* HPQL, *B. cardium* (downloaded from NCBI PRJNA490389), *B. zoohelcum* ATCC 43767, *B. zoohelcum* CCUG 30536, *B. zoohelcum* NCTC 11660 and *B. zoohelcum* NCTC 11661 genomes was carried out. Phylogenetic tree based on core genome analysis

Three genome islands were predicted from the whole genome sequences (Additional file 7 Sheet S5). No prophage was predicted from the whole genome sequence of *B. cardium* HPQL, while 3 CRISPERs sequences (Additional file 1 Table S1), 30 insertion sequences (Additional file 2 Table S2) and four toxin-antitoxin (Additional file 8 Sheet S6) were predicted from the whole genome sequence of *B. cardium* HPQL. Moreover, comparative genomic analysis between the *B. cardium* HPQL, *B. cardium* (downloaded from NCBI PRJNA490389), *B. zoohelcum* ATCC 43767, *B. zoohelcum* CCUG 30536, *B. zoohelcum* NCTC 11660 and *B. zoohelcum* NCTC 11661 genome also demonstrated the evolutionary divergence of *B. cardium* HPQL from *B. Zoohelcum* spp. (Fig. 4)*.* The relatively low sequence homology observed for the newly isolated *B. cardium* sp. with *B. zoohelcum* implied that the HPQL strain is a new member of the *Bergeyella* genus. Further comparative genomic analysis of the two *B. cardium* strains revealed 259 genes specific to *B. cardium* HPQL and 80 genes specific to another *B. cardium* strain.

### Original analysis of the *B. cardium* related to infective endocarditis

**Fig. 5:**
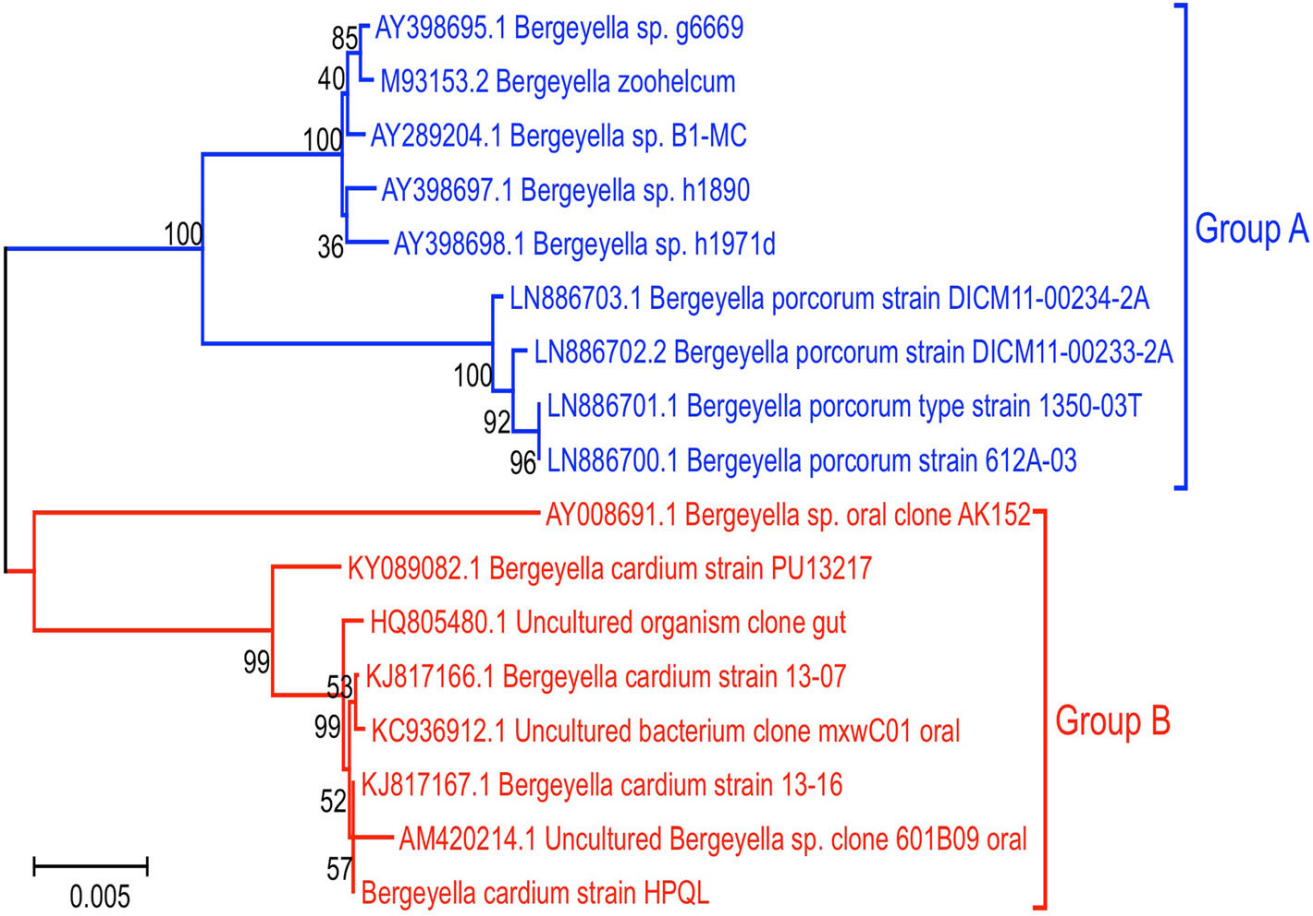
Phylogenetic tree based on 16S ribosomal DNA sequences from multiple *Bergeyella* spp. Phylogenetic analysis of *Bergeyella zoohelcum* (Group A, animal original) and (Group B, human original) homologous to *Bergeyella cardium* strain. The phylogenetic tree was created using the Neighbor-Joining algorithm. The branches are scaled in terms of the expected number of substitutions per site

To further elucidate the possible origin of the new *B.cardium* sp*.* strain, 16S rRNA sequences of the *Bergeyella* spp*.* were downloaded from NCBI for phylogenetic analysis. The analysis results demonstrated that strains homologous to *B. zoohelcum* clustered into one group, while strains homologous to the *B. cardium* strain clustered into another (Fig. 5). Interestingly, strains homologous to *B. zoohelcum* were isolated from animals, while the strains homologous to *B. cardium* were isolated from *Homo sapiens*. Moreover, two uncultured oral bacterial clones were identified with strong similarity to *B. cardium* sp., indicating that these strains also belong to the *B. cardium* sp.

## Discussion

Little is known about the genus *Bergeyella* with *B. zoohelcum* being the only well described zoonotic pathogen currently afflicting humans [6, 7]. Recently, worldwide, 4 cases of *B. cardium* sp. have been reported as being isolated from patients with infective endocarditis [3, 5, 12]. The isolates were recognized as a novel strains belonging to the genus *Bergeyella*. In this study, we reported an additional new isolate belonging to the *Bergeyella* genus, from blood cultures of infective endocarditis patients. Results from NCBI BLASTN and phylogenetic analyses reveal that the new isolate belonged to *B. cardium* (Fig. 1). The 4 previously reported cases together with our new discovery clearly suggest that *B. cardium* sp. is correlative with human infective endocarditis. However, few studies have examined the fundamental biological properties of these new strains. Herein, we provide detailed biological characterization and whole genome sequencing of the newly isolated *B. cardium* sp*.* Our study may, therefore, serve to provide fundamental information to better understand this newly identified pathogen.

Consistent with other four reported cases, our isolate was also fastidious and was found to grow slowly on blood agar. The fastidious nature of the *B. cardium* sp. may account for their rare isolation. In addition, the newly identified strain exhibited irregular rod-shaped cells similar to *B. cardium* PU13217 [12]. Biochemical analysis using API card was also carried out, which may provide better understanding of this new strain of *Bergeyella.* Furthermore, our AST results were consistent with that observed for strain 13-7^T^, demonstrating similar MIC susceptibilities in response to antimicrobial agents (Table 1). Currently there are no clearly defined standards described by NCCLS/CLSI for antibiotic susceptibility testing or breakpoints for *B. cardium*; however, our AST data, together with previous studies [3, 12], suggest effective targeted antibiotics for treatment of infections with this bacterial species.

We also sequenced the complete genome of *B. cardium* strain HPQL. To our knowledge, this is the first complete genome sequencing performed on any *Bergeyella* spp*.* Analysis results revealed 12 genes related to antibiotic resistance (Additional file 4 Sheet S2), including 3 that related to fluoroquinolone resistance, which is consistent with our in vitro AST analysis results. Sequencing results also revealed 162 genes associated with encoding virulence factors (Additional file 5 Sheet S3 and Additional file 6 Sheet S4). Identification of these genes serve to the current understanding of the mechanisms responsible for the pathogenic effects elicited by *B. cardium* strains.

The *B. cardium* strains isolated from this human patient were phylogenetically unique compared to the strains isolated from animals (Fig. 5), suggesting that the newly identified strains may originate from different sources than those that infect animals.

## Conclusions

Our data, collectively with other studies, clearly document that *B. cardium* strains are important, newly identified, human pathogens. The phylogenetic, phenotypic and morphological results together with the whole genome sequencing serve to extensively expand the current knowledge on the newly identified *Bergeyella* spp. as it relates to human infective endocarditis. Furthermore, our results provide insights into effective clinical diagnosis and treatment of this pathogen. We also suggest that this specific strain *of B. cardium* originated from the human oral cavity, though direct evidence of this was lacking. Future studies should focus on elucidating the pathogenic mechanisms elicited by this newly identified pathogen.

## Methods

### Bacteria isolation

A 63-year-old male presented to Qilu Hospital at Shandong University, Jinan, China, on April 26, 2016 with intermittent fever, fatigue, and chest distress for the previous 10 months. Ultrasonic cardiogram revealed infective endocarditis with valvular disease. Blood samples were sent to the microbiology laboratory for culturing on April 30, 2016. The blood cultures were incubated in the Bactec system (Becton Dickinson, Franklin Lakes, NJ) until a positive result was obtained. The positive blood cultures were inoculated onto Columbia blood agar, MacConkey agar, and Chocolate agar and incubated (Thermo Fisher Scientific, USA) at 35 °C until visible colonies appeared. Colonies were purified using blood agar for further analysis.

### Phylogenetic analysis

The nucleotide sequences of 16S rRNA genes from different bacterial strains were downloaded from the NCBI database (http://www.ncbi.nlm.nih.gov) and aligned using the ClustalX computer program. The aligned sequences were refined and phylogenetically analyzed using distance/neighbor joining (NJ) and maximum-likelihood (ML) algorithms with the Poisson correction distance model in the MEGA software package [13] to infer their phylogenetic relationships. The bootstrapping supports for the interior branch length of the trees were from 1000 replicates.

### Morphological, physiological and biochemical characterization

Morphological characterization of the isolated bacterial strain was carried out as previously described [14]. Growth was examined on Columbia blood, MacConkey and Chocolate agar. The strain was further biochemically characterized using API NH card (bioMérieux, Marcy l’Étoile, France) according to the manufacturer’s instructions.

### Antibiotic sensitivity analysis

Both the Vitek 2 system (bioMérieux, Marcy l’Étoile, France) and PDM Epsilometer test (E test) were employed to determine the antibiotic susceptibility of the isolated strain. For the Vitek 2 system, the cell density of the bacterial colony was adjusted to a density of 0.5 McFarland with 0.45% saline; 145 μL of the bacterial suspension was then added into 3 mL of 0.45% saline solution to further adjust the bacterial cell density. The suspension vials were then applied to the Vitek GN09 card and loaded into the Vitek 2 automated reader-incubator for analysis. For the E test, the 0.5 McFarland bacterial cell suspension were surface plated onto Blood agar plates, using a sterile swab to produce an even inoculum [15]. The plates were then incubated for 96 h (Thermo Fisher Scientific, USA) at 35 °C. The minimum inhibitory concentration (MIC) was determined to be the point where the elliptical zone of growth inhibition intersected with the MIC scale on the E test strip [15]. Sensitivities to penicillin, ceftriaxone, cefepime, cefotaxime, meropenem, imipenem, tigecycline, amoxicillin/clavulanate potassium, sulfamethoxazole, levofloxacin, ciprofloxacin, chloramphenicol, azithromycin, and gentamycin were examined.

### Genome sequencing and assembly

Genomic sequencing and assembly were carried out at Novogen Bioinformatics Technology Co., Ltd. (Beijing, China). Single-molecule real-time (SMRT®) sequencing was performed using a Pacific Biosciences RSII sequencer (PacBio, Menlo Park, CA) according to the manufacturer’s instructuions (MagBead Standard Seq v2 loading, 1 × 180 min movie) using P4-C2 chemistry. The low-quality reads were filtered by the SMRT 2.3.0 and the filtered reads were then assembled to generate one contig without gaps. Hierarchical Genome Assembly Process (HGAP) pipeline was used for the whole genome assemble.

### Genome annotations

The assembled genome sequence was annotated further. Small RNAs (sRNAs) were predicted by BLAST against the Rfam [16] database. tRNAscan-SE [17] was then used to predicted transfer RNA (tRNA) genes, while the rRNAmmer server [18] was used to predict ribosomal RNA (rRNA) genes. RepeatMasker [19] and Tandem Repeat Finder [20] were applied to predict repetitive sequences and tandem repeats, respectively. A whole genome alignment (E-value less than 1e-5 and a minimal alignment length percentage > 40%) against 6 databases, namely Clusters of Orthologous Groups (COG), Kyoto Encyclopedia of Genes and Genomes (KEGG), NCBI non-redundant (NR), Swiss-Prot, Gene Ontology (GO) and Translated EMBL (TrEMBL) was performed to predict gene functions [21–27]. ISFinder blast (https://www-is.biotoul.fr/blast.php) was used to predicted IS sequences while CRISPRdigger (https://omictools.com/crisprdigger-tool) [28] was used to predict CRISPR sequences. Prophage was predicted using PHASTER (http://phaster.ca) and IslandPath-DIOMB [29] was used to predict genome islands. RASTA-Bacteria (http://genoweb1.irisa.fr/duals/RASTA-Bacteria/) was used to identify toxin-antitoxins. The methylation data had been submitted to REBASE database for restriction modification system analysis.

### Prediction of genes related to antibiotic resistance and virulence factors

The genome sequences of the HPQL bacterial strain were submitted to the Virulence Factors of Pathogenic Bacteria (VFDB) [30], Comprehensive Antibiotic Research Database (CARD) [31] and Pathogen-Host Interactions database (PHI) [32] databases to predict which genes were related to antibiotic resistance and virulence factors.

### Comparative genomics analysis

Comparative genomic analysis was performed between the *B. cardium* HPQL genome, *B. cardium* (downloaded from NCBI PRJNA490389) *B. zoohelcum* ATCC 43767 genome (downloaded from NCBI), the *B. zoohelcum* CCUG 30536 genome (downloaded from NCBI), *B. zoohelcum* NCTC 11660 genome (downloaded from NCBI), and *B. zoohelcum* NCTC 11661 (downloaded from NCBI). Core genes and specific genes were analyzed via CD-HIT rapid clustering of similar proteins software with the threshold set to 50% pairwise identity and a 0.7 cutoff in length difference of amino acids [25, 33, 34]. A phylogenetic tree was also constructed using the TreeBeST [35] according to the PhyML method, and the setting of bootstraps was 1,000 with the orthologous genes.

### Nucleotide sequence accession numbers

The obtained genome sequence for *B. cardium* HPQL was deposited in GenBank under the accession numbers CP029149.

## Supplementary information

**Additional file 1: Table S1.** The predicted CRISPR sequences in the genome of *B. cardium* HPQL.

**Additional file 2: Table S2.** The IS sequences in the genome of *B. cardium* HPQL.

**Additional file 3:** The methylation data of the whole genome.

**Additional file 4:** The predicted genes related to antibiotic resistance.

**Additional file 5:** Genes related to putative virulence factors.

**Additional file 6:** Genes related to bacteria-host interactions.

**Additional file 7:** The predicted genome islands

**Additional file 8:** The predicted toxin-antitoxins

## Data Availability

The obtained genome sequence for *B. cardium* HPQL was deposited in GenBank under the accession numbers CP029149. In addition, the methylation data of the whole genome, whole genome sequences and predicted restriction modification system were also available in REBASE database (http://rebase.neb.com/rebase/private/pacbio_Pan15.html). All data generated or analysed during this study are included in this published article and its supplementary information files. The datasets used and/or analysed during the current study are also available from the corresponding author on reasonable request.

## Abbreviations

*B. cardium*: *Bergeyella cardium*
*CARD*: Comprehensive Antibiotic Research Database
CDS: Coding sequences
COG: Clusters of Orthologous Group
ESR: Erythrocyte sedimentation rate
GO: Gene Ontology
HPQL: Hongwei Pan from QiLu hospital
KEGG: Kyoto Encyclopedia of Genes and Genomes
MIC: Minimum inhibitory concentration
ML: Maximum likelihood
NJ: Neighbor joining
NR: NCBI non-redundant database
PHI: Pathogen-Host Interactions database
TrEMBL: Translated EMBL
VFDB: Virulence Factors of Pathogenic Bacteria
