# Supplementary material for: Characterization and whole genome sequencing of a novel strain of Bergeyella cardium related to infective endocarditis
Source: BMC Microbiol. 2020 Feb 12;20:32. doi: 10.1186/s12866-020-1715-0 (PMC7017618; doi:10.1186/s12866-020-1715-0)
Supplement: Supplementary file 1 — Additional file 1: Table S1. The predicted CRISPR sequences in the genome of B. cardium HPQL. [file 12866_2020_1715_MOESM1_ESM.docx]

**Table S1: The predicted CRISPR sequences in the genome of *B. cardium* HPQL**

| **ID** | **Location** | **Strand** | **Length** | **aSPLength** | **SPnum** | **aDRLength** |
| --- | --- | --- | --- | --- | --- | --- |
| **Crispr 1** | **1504038-1506151** | **-** | **2114** | **30** | **27** | **46** |
| **Crispr 2** | **1972098-1972352** | **-** | **255** | **48** | **3** | **27** |
| **Crispr 3** | **1974327-1974581** | **-** | **255** | **45** | **3** | **30** |
