# Supplementary material for: Characterization and whole genome sequencing of a novel strain of Bergeyella cardium related to infective endocarditis
Source: BMC Microbiol. 2020 Feb 12;20:32. doi: 10.1186/s12866-020-1715-0 (PMC7017618; doi:10.1186/s12866-020-1715-0)
Supplement: Supplementary file 2 — Additional file 2: Table S2. The IS sequences in the genome of B. cardium HPQL. [file 12866_2020_1715_MOESM2_ESM.docx]

**Table S2: The IS sequences in the genome of *B. cardium* HPQL**

| **Sequences producing significant alignments** | **IS Family** | **Group** | ***Origin*** | **Score (bits)** | **E value** |
| --- | --- | --- | --- | --- | --- |
| [ISAur1](https://www-is.biotoul.fr/scripts/ficheIS.php?name=ISAur1) | IS1595 | ISPna2 | *[Actinobacillus ureae](http://www.ncbi.nlm.nih.gov/Taxonomy/Browser/wwwtax.cgi?name=Actinobacillus+ureae)* | [67.9](https://www-is.biotoul.fr/blast/resultat.php?id=phpAEI3aX&title=&prog=blastn#BL_ORD_ID:4856) | 6e-08 |
| [ISMma2](https://www-is.biotoul.fr/scripts/ficheIS.php?name=ISMma2) | IS1182 |  | *[Methanosarcina mazei](http://www.ncbi.nlm.nih.gov/Taxonomy/Browser/wwwtax.cgi?name=Methanosarcina+mazei)* | [50.1](https://www-is.biotoul.fr/blast/resultat.php?id=phpAEI3aX&title=&prog=blastn#BL_ORD_ID:2725) | 0.014 |
| [ISMac1](https://www-is.biotoul.fr/scripts/ficheIS.php?name=ISMac1) | IS1182 |  | *[Methanosarcina acetivorans](http://www.ncbi.nlm.nih.gov/Taxonomy/Browser/wwwtax.cgi?name=Methanosarcina+acetivorans)* | [50.1](https://www-is.biotoul.fr/blast/resultat.php?id=phpAEI3aX&title=&prog=blastn#BL_ORD_ID:2724) | 0.014 |
| [ISSulsp1](https://www-is.biotoul.fr/scripts/ficheIS.php?name=ISSulsp1) | IS5 |  | *[Sulfurihydrogenibium sp.](http://www.ncbi.nlm.nih.gov/Taxonomy/Browser/wwwtax.cgi?name=Sulfurihydrogenibium+sp.)* | [48.1](https://www-is.biotoul.fr/blast/resultat.php?id=phpAEI3aX&title=&prog=blastn#BL_ORD_ID:3528) | 0.056 |
| [ISMac2](https://www-is.biotoul.fr/scripts/ficheIS.php?name=ISMac2) | IS1182 |  | *[Methanosarcina acetivorans](http://www.ncbi.nlm.nih.gov/Taxonomy/Browser/wwwtax.cgi?name=Methanosarcina+acetivorans)* | [46.1](https://www-is.biotoul.fr/blast/resultat.php?id=phpAEI3aX&title=&prog=blastn#BL_ORD_ID:2726) | 0.22 |
| [ISChpi1](https://www-is.biotoul.fr/scripts/ficheIS.php?name=ISChpi1) | IS1595 |  | *[Chryseobacterium piperi](http://www.ncbi.nlm.nih.gov/Taxonomy/Browser/wwwtax.cgi?name=Chryseobacterium+piperi)* | [44.1](https://www-is.biotoul.fr/blast/resultat.php?id=phpAEI3aX&title=&prog=blastn#BL_ORD_ID:4875) | 0.87 |
| [ISCpe5](https://www-is.biotoul.fr/scripts/ficheIS.php?name=ISCpe5) | IS1182 |  | *[Clostridium perfringens](http://www.ncbi.nlm.nih.gov/Taxonomy/Browser/wwwtax.cgi?name=Clostridium+perfringens)* | [44.1](https://www-is.biotoul.fr/blast/resultat.php?id=phpAEI3aX&title=&prog=blastn#BL_ORD_ID:2751) | 0.87 |
| [ISSc1](https://www-is.biotoul.fr/scripts/ficheIS.php?name=ISSc1) | IS30 |  | *[Spiroplasma citri](http://www.ncbi.nlm.nih.gov/Taxonomy/Browser/wwwtax.cgi?name=Spiroplasma+citri)* | [44.1](https://www-is.biotoul.fr/blast/resultat.php?id=phpAEI3aX&title=&prog=blastn#BL_ORD_ID:699) | 0.87 |
| [ISRa1](https://www-is.biotoul.fr/scripts/ficheIS.php?name=ISRa1) | IS982 |  | *[Riemerella anati](http://www.ncbi.nlm.nih.gov/Taxonomy/Browser/wwwtax.cgi?name=Riemerella+anati)* | [44.1](https://www-is.biotoul.fr/blast/resultat.php?id=phpAEI3aX&title=&prog=blastn#BL_ORD_ID:631) | 0.87 |
| [ISLbp1](https://www-is.biotoul.fr/scripts/ficheIS.php?name=ISLbp1) | IS4 | IS4 | *[Leptospira borgpetersenii](http://www.ncbi.nlm.nih.gov/Taxonomy/Browser/wwwtax.cgi?name=Leptospira+borgpetersenii)* | [42.1](https://www-is.biotoul.fr/blast/resultat.php?id=phpAEI3aX&title=&prog=blastn#BL_ORD_ID:5406) | 3.4 |
| [ISPa90](https://www-is.biotoul.fr/scripts/ficheIS.php?name=ISPa90) | IS1182 |  | *[Pseudomonas aeruginosa](http://www.ncbi.nlm.nih.gov/Taxonomy/Browser/wwwtax.cgi?name=Pseudomonas+aeruginosa)* | [42.1](https://www-is.biotoul.fr/blast/resultat.php?id=phpAEI3aX&title=&prog=blastn#BL_ORD_ID:5203) | 3.4 |
| [ISEisp1](https://www-is.biotoul.fr/scripts/ficheIS.php?name=ISEisp1) | IS1595 | ISPna2 | *[Eikenella sp.](http://www.ncbi.nlm.nih.gov/Taxonomy/Browser/wwwtax.cgi?name=Eikenella+sp.)* | [42.1](https://www-is.biotoul.fr/blast/resultat.php?id=phpAEI3aX&title=&prog=blastn#BL_ORD_ID:4861) | 3.4 |
| [ISClsp5](https://www-is.biotoul.fr/scripts/ficheIS.php?name=ISClsp5) | IS1595 | ISPna2 | *[Clostridium sp.](http://www.ncbi.nlm.nih.gov/Taxonomy/Browser/wwwtax.cgi?name=Clostridium+sp.)* | [42.1](https://www-is.biotoul.fr/blast/resultat.php?id=phpAEI3aX&title=&prog=blastn#BL_ORD_ID:4846) | 3.4 |
| [ISTme1](https://www-is.biotoul.fr/scripts/ficheIS.php?name=ISTme1) | IS1595 | ISPna2 | *[Thalassobius mediterraneus](http://www.ncbi.nlm.nih.gov/Taxonomy/Browser/wwwtax.cgi?name=Thalassobius+mediterraneus)* | [42.1](https://www-is.biotoul.fr/blast/resultat.php?id=phpAEI3aX&title=&prog=blastn#BL_ORD_ID:4821) | 3.4 |
| [ISHahy2](https://www-is.biotoul.fr/scripts/ficheIS.php?name=ISHahy2) | IS3 | IS150 | *[Halanaerobium hydrogeniformans](http://www.ncbi.nlm.nih.gov/Taxonomy/Browser/wwwtax.cgi?name=Halanaerobium+hydrogeniformans)* | [42.1](https://www-is.biotoul.fr/blast/resultat.php?id=phpAEI3aX&title=&prog=blastn#BL_ORD_ID:4669) | 3.4 |
| [ISAfe13](https://www-is.biotoul.fr/scripts/ficheIS.php?name=ISAfe13) | ISKra4 | ISAzba1 | *[Acidithiobacillus ferrivorans](http://www.ncbi.nlm.nih.gov/Taxonomy/Browser/wwwtax.cgi?name=Acidithiobacillus+ferrivorans)* | [42.1](https://www-is.biotoul.fr/blast/resultat.php?id=phpAEI3aX&title=&prog=blastn#BL_ORD_ID:3946) | 3.4 |
| [ISCla2](https://www-is.biotoul.fr/scripts/ficheIS.php?name=ISCla2) | ISLre2 |  | *[Caldicellulosiruptor lactoaceticus](http://www.ncbi.nlm.nih.gov/Taxonomy/Browser/wwwtax.cgi?name=Caldicellulosiruptor+lactoaceticus)* | [42.1](https://www-is.biotoul.fr/blast/resultat.php?id=phpAEI3aX&title=&prog=blastn#BL_ORD_ID:3875) | 3.4 |
| [ISCbo1](https://www-is.biotoul.fr/scripts/ficheIS.php?name=ISCbo1) | IS6 |  | *[Clostridium botulinum](http://www.ncbi.nlm.nih.gov/Taxonomy/Browser/wwwtax.cgi?name=Clostridium+botulinum)* | [42.1](https://www-is.biotoul.fr/blast/resultat.php?id=phpAEI3aX&title=&prog=blastn#BL_ORD_ID:3639) | 3.4 |
| [ISCra1](https://www-is.biotoul.fr/scripts/ficheIS.php?name=ISCra1) | IS1380 |  | *[Cylindrospermopsis raciborskii](http://www.ncbi.nlm.nih.gov/Taxonomy/Browser/wwwtax.cgi?name=Cylindrospermopsis+raciborskii)* | [42.1](https://www-is.biotoul.fr/blast/resultat.php?id=phpAEI3aX&title=&prog=blastn#BL_ORD_ID:2821) | 3.4 |
| [ISSau8](https://www-is.biotoul.fr/scripts/ficheIS.php?name=ISSau8) | ISL3 |  | *[Staphylococcus aureus](http://www.ncbi.nlm.nih.gov/Taxonomy/Browser/wwwtax.cgi?name=Staphylococcus+aureus)* | [42.1](https://www-is.biotoul.fr/blast/resultat.php?id=phpAEI3aX&title=&prog=blastn#BL_ORD_ID:1728) | 3.4 |
| [IS712](https://www-is.biotoul.fr/scripts/ficheIS.php?name=IS712) | IS21 |  | *[Lactococcus lactis](http://www.ncbi.nlm.nih.gov/Taxonomy/Browser/wwwtax.cgi?name=Lactococcus+lactis)* | [42.1](https://www-is.biotoul.fr/blast/resultat.php?id=phpAEI3aX&title=&prog=blastn#BL_ORD_ID:1681) | 3.4 |
| [ISCbe4](https://www-is.biotoul.fr/scripts/ficheIS.php?name=ISCbe4) | ISLre2 |  | *[Caldicellulosiruptor bescii](http://www.ncbi.nlm.nih.gov/Taxonomy/Browser/wwwtax.cgi?name=Caldicellulosiruptor+bescii)* | [42.1](https://www-is.biotoul.fr/blast/resultat.php?id=phpAEI3aX&title=&prog=blastn#BL_ORD_ID:1421) | 3.4 |
| [ISNpu4](https://www-is.biotoul.fr/scripts/ficheIS.php?name=ISNpu4) | IS4 | IS10 | *[Nostoc punctiforme](http://www.ncbi.nlm.nih.gov/Taxonomy/Browser/wwwtax.cgi?name=Nostoc+punctiforme)* | [42.1](https://www-is.biotoul.fr/blast/resultat.php?id=phpAEI3aX&title=&prog=blastn#BL_ORD_ID:1374) | 3.4 |
| [ISFac8](https://www-is.biotoul.fr/scripts/ficheIS.php?name=ISFac8) | IS256 |  | *[Ferroplasma acidarmanus](http://www.ncbi.nlm.nih.gov/Taxonomy/Browser/wwwtax.cgi?name=Ferroplasma+acidarmanus)* | [42.1](https://www-is.biotoul.fr/blast/resultat.php?id=phpAEI3aX&title=&prog=blastn#BL_ORD_ID:1108) | 3.4 |
| [ISMac13](https://www-is.biotoul.fr/scripts/ficheIS.php?name=ISMac13) | IS630 |  | *[Methanosarcina acetivorans](http://www.ncbi.nlm.nih.gov/Taxonomy/Browser/wwwtax.cgi?name=Methanosarcina+acetivorans)* | [42.1](https://www-is.biotoul.fr/blast/resultat.php?id=phpAEI3aX&title=&prog=blastn#BL_ORD_ID:1063) | 3.4 |
| [ISSau5](https://www-is.biotoul.fr/scripts/ficheIS.php?name=ISSau5) | IS30 |  | *[Staphylococcus aureus](http://www.ncbi.nlm.nih.gov/Taxonomy/Browser/wwwtax.cgi?name=Staphylococcus+aureus)* | [42.1](https://www-is.biotoul.fr/blast/resultat.php?id=phpAEI3aX&title=&prog=blastn#BL_ORD_ID:940) | 3.4 |
| [ISMi1](https://www-is.biotoul.fr/scripts/ficheIS.php?name=ISMi1) | IS3 | IS150 | *[Mycoplasma incognitus](http://www.ncbi.nlm.nih.gov/Taxonomy/Browser/wwwtax.cgi?name=Mycoplasma+incognitus)* | [42.1](https://www-is.biotoul.fr/blast/resultat.php?id=phpAEI3aX&title=&prog=blastn#BL_ORD_ID:590) | 3.4 |
| [IS656](https://www-is.biotoul.fr/scripts/ficheIS.php?name=IS656) | IS1182 |  | *[Bacillus halodurans](http://www.ncbi.nlm.nih.gov/Taxonomy/Browser/wwwtax.cgi?name=Bacillus+halodurans)* | [42.1](https://www-is.biotoul.fr/blast/resultat.php?id=phpAEI3aX&title=&prog=blastn#BL_ORD_ID:411) | 3.4 |
| [IS4Sa](https://www-is.biotoul.fr/scripts/ficheIS.php?name=IS4Sa) | IS4 | IS4Sa | *[Synechocystis sp.](http://www.ncbi.nlm.nih.gov/Taxonomy/Browser/wwwtax.cgi?name=Synechocystis+sp.)* | [42.1](https://www-is.biotoul.fr/blast/resultat.php?id=phpAEI3aX&title=&prog=blastn#BL_ORD_ID:367) | 3.4 |
| IS1550 | IS3 | IS150 | *[Mycoplasma fermentans](http://www.ncbi.nlm.nih.gov/Taxonomy/Browser/wwwtax.cgi?name=Mycoplasma+fermentans)* | 42.1 | 3.4 |
